# Metal and Precursor Effect during 1-Heptyne Selective Hydrogenation Using an Activated Carbon as Support

**DOI:** 10.1155/2013/528453

**Published:** 2013-11-14

**Authors:** Cecilia R. Lederhos, Juan M. Badano, Nicolas Carrara, Fernando Coloma-Pascual, M. Cristina Almansa, Domingo Liprandi, Mónica Quiroga

**Affiliations:** ^1^INCAPE, Instituto de Investigaciones en Catálisis y Petroquímica (FIQ-UNL, CONICET), Santiago del Estero 2654, 3000 Santa Fe, Argentina; ^2^Servicios Técnicos de Investigación, Facultad de Ciencias, Universidad de Alicante, Apartado 99, 03080 Alicante, Spain; ^3^Facultad de Ingenería Química, Universidad Nacional del Litoral, Santiago del Estero 2829, 3000 Santa Fe, Argentina

## Abstract

Palladium, platinum, and ruthenium supported on activated carbon were used as catalysts for the selective hydrogenation of 1-heptyne, a terminal alkyne. All catalysts were characterized by temperature programmed reduction, X-ray diffraction, transmission electron microscopy, and X-ray photoelectron spectroscopy. TPR and XPS suggest that the metal in all catalysts is reduced after the pretreatment with H_2_ at 673 K. The TPR trace of the PdNRX catalyst shows that the support surface groups are greatly modified as a consequence of the use of HNO_3_ during the catalyst preparation. During the hydrogenation of 1-heptyne, both palladium catalysts were more active and selective than the platinum and ruthenium catalysts. The activity order of the catalysts is as follows: PdClRX > PdNRX > PtClRX ≫ RuClRX. This superior performance of PdClRX was attributed in part to the total occupancy of the d electronic levels of the Pd metal that is supposed to promote the rupture of the H_2_ bond during the hydrogenation reaction. The activity differences between PdClRX and PdNRX catalysts could be attributed to a better accessibility of the substrate to the active sites, as a consequence of steric and electronic effects of the superficial support groups. The order for the selectivity to 1-heptene is as follows: PdClRX = PdNRX > RuClRX > PtClRX, and it can be mainly attributed to thermodynamic effects.

## 1. Introduction

Carbonaceous materials are widely used as catalyst supports in industrial reactions, especially for hydrogenation and hydrodechlorination processes [1–3]. The catalysts based on activated carbon (AC) have several advantages in comparison to silica and alumina-supported catalysts, for example, low cost, stability, high superficial area, and inertness in liquid reaction media. On the other hand, catalysts prepared using AC generally present low deactivation; it is possible to change the chemical surface of the carbon during the catalyst preparation and the recovery of the metal phase from the spent catalysts is easy [4, 5]. It is also well known that treatments with acidic solutions (of the precursor salts) as well as reduction treatments with hydrogen can modify the properties of the active carbons [6, 7].

In recent years, the catalytic hydrogenation of acetylenes has been widely studied due to its academic and industrial interest. It is necessary that the catalyst does not promote over hydrogenation, thus forming the corresponding alkane. The selective hydrogenation of an alkyne to the corresponding alkene over metallic catalysts is possible because the alkyne is more strongly bonded to the catalytic active sites, thus competing with the alkene, limiting its readsorption and its undesirable hydrogenation. The obtained products from this type of reactions are very important in the synthesis of active biological compounds, being also the raw material for different industrial processes like production of margarine, lubricants, and so forth. Additionally, they are an important tool for several reactions in fine chemistry. A survey of the scientific literature indicates that practically all studies were concentrated on the reaction of short chain terminal alkynes over supports such as Al_2_O_3_, SiO_2_, or CaCO_3_ [8–17]. Scarce literature is devoted to the use of carbonaceous catalysts for the selective hydrogenation of long-chain alkynes [18–23].

Based on the above considerations, the objectives of this work were (i) to prepare and characterize low-loaded catalysts with different precursor metals on AC and (ii) to evaluate the activity and selectivity of the catalysts during the partial hydrogenation of 1-heptyne at mild conditions.

## 2. Experimental

### 2.1. Catalyst Preparation

Activated carbon RX 3 EXTRA powdered and provided by NORIT was used as support (*S*_BET_: 1398 m^2^ g^−1^ and pore volume 0.69 cm^3^ g^−1^). Acidic solutions (pH = 1) of PdCl_2_, Pd(NO_3_)_2_·2H_2_O, H_2_PtCl_6_, and RuCl_3_ (with HCl or HNO_3_, according to the precursor salt) were used for impregnation of the support by means of the incipient wetness technique. After the impregnation with the acidic solutions, all the catalysts were dried overnight at 373 K and treated in flowing hydrogen at 673 K for 1 h.

### 2.2. Catalyst Characterization

The chemical composition of the final catalysts was determined by ICP in an OPTIMA 21200 Perkin Elmer equipment. The samples were dissolved with dilute solution of sulfuric acid at 363 K before each analysis. 

Brunauer-Emmett-Teller surface area (*S*_BET_) of the prepared catalysts was determined by means of nitrogen physisorption at 77 K following the BET model in a Quantachrome Corporation NOVA-1000 equipment. Before the measurement, the samples were degassed at 573 K in vacuum.

The particle size of the supported metals was studied by transmission electronic microscopy (TEM) using an electronic microscope JEOL JEM-2010 at 200 kV.

The metals reducibility was determined by temperature programmed reduction (TPR) using a Micromeritics AutoChem II 2920 instrument equipped with a thermal conductivity detector. The samples were treated at 373 K for 30 min under an argon stream in order to eliminate humidity; the samples were cooled down to room temperature and finally heated up to 723 K at 10 K min^−1^ in a 5 (% v/v) hydrogen in argon gas stream.

The electronic state of the metals was studied by X-ray photoelectron spectroscopy (XPS) by inspection of the Pd 3d_5/2_, Pt 4f_7/2_, and Ru 3d_5/2_ peaks. Measurements were done using a VG-Microtech Multilab equipment, with a MgK_*α*_ (*hν*: 1253.6 eV) radiation source and a pass energy of 50 eV. The XPS system analysis pressure was kept at 5.10^−7 ^Pa. Samples were treated *in situ* 1 h with H_2_ at 673 K. The areas of the peaks were estimated by calculating the integral of each peak after subtracting a Shirley background and fitting the experimental peak to a combination of Lorentzian/Gaussian lines of 30–70% proportions. The reference binding energy (BE) was the C1s peak at 284.5 eV. A careful deconvolution of the spectra was made. Determinations of the superficial atomic ratios were made by comparing the normalized areas under the peaks after background subtraction and corrections due to differences in escape depth and in photoionization cross-sections [24].

X-ray diffraction (XRD) measurements of powdered samples were obtained using a Shimadzu XD-D1 instrument with CuK_*α*_ radiation (*λ* = 1.5405 Å) in the 20° < 2*θ* < 80° at a scan rate of 0.5° min^−1^.

### 2.3. Catalytic Evaluation

The catalytic evaluations were performed in a batch stainless steel stirred tank reactor equipped with a magnetically driven stirrer. The stirrer had two blades in counter rotation and was operated at 800 rpm. The inner wall of the reactor was completely coated with PTFE in order to neglect the catalytic action of the steel of the reactor, as found by other authors [25]. The possibility of diffusional limitations during the catalytic tests was investigated by means of procedures previously described [26]. Experiments were carried out at different stirring rates in the 180–1400 rpm range. The constancy of the activity and selectivity above 500 rpm ensured that external diffusional limitations were absent at the rotary speed selected (800 rpm). Runs were carried out in triplicates with an experimental error of 3%.

Selective hydrogenation of 1-heptyne was carried out at 303 K using 0.3 g of catalyst and an alkyne/M molar ratio (M = Pd, Pt, or Ru) that is equal to 2000. The hydrogen pressure in all the experiments was 150 kPa because it is well established in the literature that high alkene selectivity values require low hydrogen pressures [27]. 75 mL of a 5% (v/v) solution of 1-heptyne (Fluka, purity > 98%) in toluene (Merck, purity > 99%) was used as feed. Reactant and products were analyzed by gas chromatography using a flame ionization detector and a capillary column.

## 3. Results and Discussion

Table 1 presents the catalyst properties as obtained by ICP, nitrogen physisorption and XPS. The BET surface area of the support is also included in Table 1 for the sake of comparison. It can be seen that all catalysts have lower values of *S*_BET_ than those of the support, suggesting that the metal particles are blocking at least partly the total surface area (17–23%).

In a previous work of our group [28], it was found that it is possible to modify the concentration of oxygenated surface groups using different acidic solutions during the preparation step. This changed the adsorption properties of the carbons and caused steric effects on the deposition of metals. The increment in the quantity of superficial groups produced by treatment with HNO_3_ produced (i) the wetting of the carbon pores with polar solvents thus increasing the effective accessible surface of the support for the metal hydroxides and (ii) a chemical interaction with the metal particles [29]. Durán-Valle et al. [30] found that RX3 carbon pretreated with nitric acid decreases the hydrogen loading with respect to the activated carbon without pretreatment. This is associated to the loss of more reactive aliphatic chains, which are replaced by heteroatoms from the oxoacid. Other authors [31] suggest that the pretreatment of activated carbon with a low concentration of HNO_3_ (as in this work) removes the impurities blocking the pore channels. On one hand, this improves the structure properties of the carbon. While on the other hand, the formation of oxygen-containing groups by HNO_3_ treatment partially blocks the micropore mouths.

**Figure 1:**
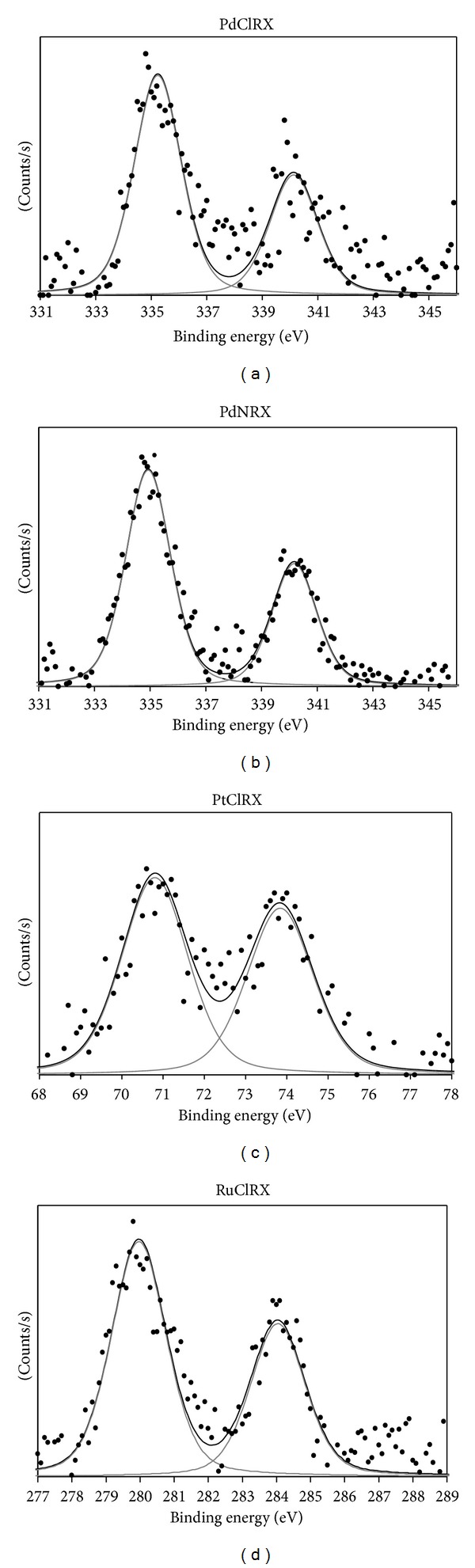
XPS spectra of PdClRX, PdNRX, PtClRX, and RuClRX catalysts treated with H_2_ at 673 K.

Figure 1 shows the XPS spectra of PdClRX, PdNRX, PtClRX, and RuClRX after deconvolution. The Pd 3d_5/2_, Pt 4f_7/2_, and Ru 3d_5/2_ binding energy (BE) values are informed in Table 1. For the PdClRX and PdNRX samples, the Pd 3d_5/2_ peak appeared at 335.3 and 334.9 eV, respectively. According to the bibliography [32], these signals could be attributed, respectively, to Pd^*δ*+^ (*δ*≅0) and Pd° species present on the catalyst surface after the reduction pretreatment at 673 K for both catalysts. The Pt 4f_7/2_ and Ru 3d_5/2_ BE peaks were 70.8 and 280.0 eV for the PtClRX and RuClRX, respectively. These values can be attributed to Pt° and Ru° species on the surface of each reduced catalyst [32]. 

**Figure 2:**
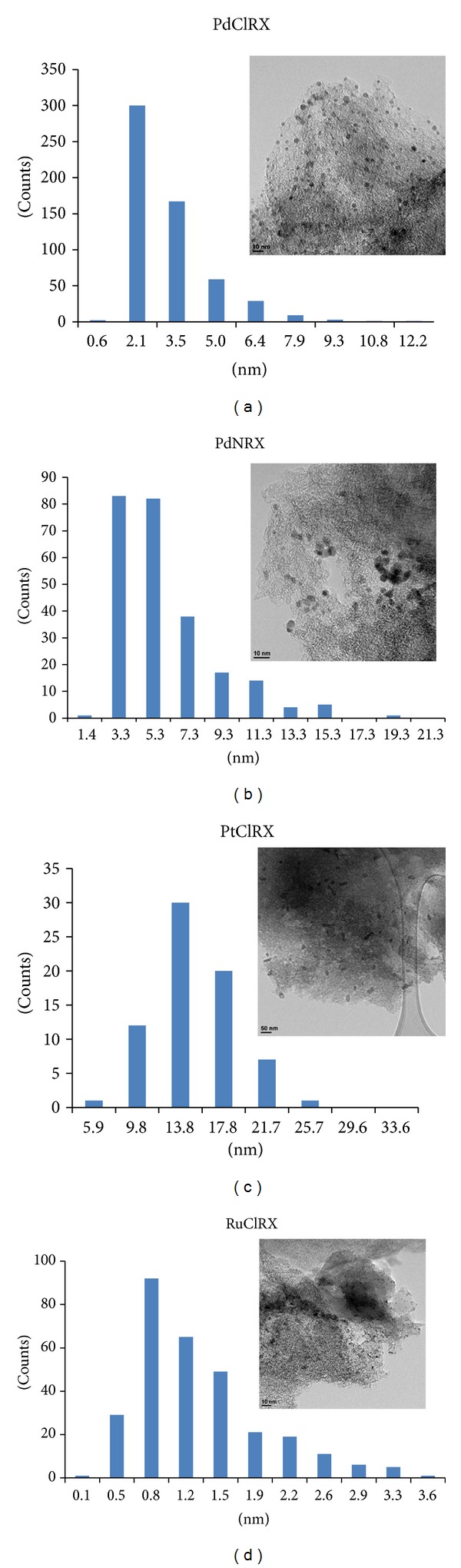
Particle size distribution and TEM images of PdClRX, PdNRX, PtClRX, and RuClRX.

Plots of the particle size distribution and TEM micrographs are shown in Figure 2. For all the catalysts, the high percentage metal particle sizes are listed in Table 1. The metal particle size for the PdClRX catalyst is between 2.1 and 7.9 nm, presenting a high concentration of 2.1 nm Pd particles on the carbon surface. For PdNRX, the main palladium particle size is between 3.3 and 5.3 nm, even though the metal particles in this catalyst are found to be up to 15.3 nm. The found particle size distribution is in total accordance with the results obtained by other authors for 1 wt% Pd supported on RX3 carbon by means of the incipient wetness technique [27, 33]. Platinum particle sizes on PtClRX catalyst are mainly between 13.8 and 17.8 nm. Besides, ruthenium catalyst has the smallest particle size, between 0.45 and 2.57 nm, with particles smaller than 2 nm.

**Figure 3:**
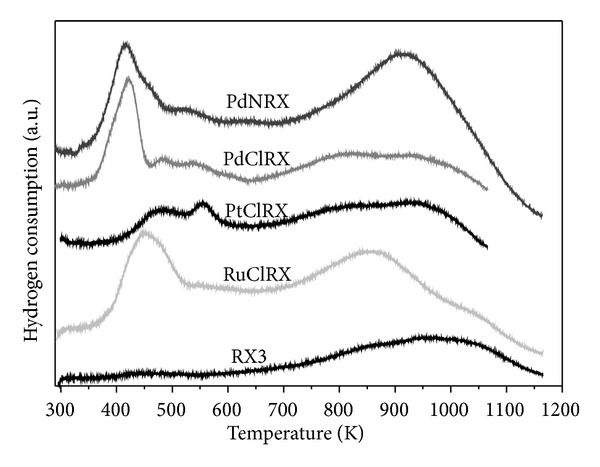
TPR profiles for PdClRX, PdNRX, PtClRX, and RuClRX catalysts and RX3 support.

In Figure 3, the TPR profiles of the PdClRX, PdNRX, PtClRX, and RuClRX catalysts are plotted. The TPR trace of the carbonaceous support (RX3) is also presented in Figure 3. It has a broad reduction peak above 700 K. A similar behavior was found for all the catalysts at high temperatures. The carbon surface is usually complex, presenting several groups, specially oxygenated ones, like phenols, carbonyls, carboxylic, and so forth, and also nitrogen groups which can be modified during the thermal pretreatments steps, either in the presence or absence of hydrogen. The peak above 700 K for sample RX3 can be attributed to the generation of CO and CO_2_ due to carbon gasification [34] or to the reduction of oxygenated groups of the support or due to the possible presence of impurities (5-6%) [35]. In a previous paper, Figueiredo et al. [36] using TPD analysis showed that if the activated carbon is treated with an acidic oxidant medium during the preparation step, the concentration of carboxylic groups on the support is increased. Li et al. [37] observed that most of the functional groups can be generated by nitric acid treatment. In Figure 3, it can be seen that the peak above 700 K is more intense for PdNRX and RuClRX catalysts, while it is rather similar for PdClRX and PtClRX catalysts. According to the literature, the presence of oxidized species on the carbon supports, carbonyl groups, phenols, and quinones, which are reduced at high temperatures, is well known [38]. Therefore, it can be concluded that the peak above 700 K can be associated with the reduction of oxidized species present on the carbon surface. Also, it can be seen in Figure 3 that the trace of the PdNRX catalyst exhibits a peak above 700 K with a higher intensity than the other catalysts, indicating a substantial modification of the surface groups of the support in this catalyst as originated by the treatment with HNO_3_.

As it can be seen in Figure 3, at lower temperatures, the traces of the palladium monometallic catalysts (PdNRX and PdClRX) are quite similar. These catalysts present a main reduction peak at 420 K and three peaks of lower intensity at 470, 529, and 610 K. These peaks could be mainly attributed to the reduction of Pd^2+^ species interacting strongly with the surface of the carbonaceous support [39–41]. For the Pd catalysts, the presence of several reduction peaks and the observed differences in the peak positions are an indication of the reduction of oxygenated surface species: the dissociative chemisorbed hydrogen over the metal can move by a spillover phenomenon on the support surface to sites containing oxygenated or chlorinated chemisorbed species, and thus competing with the reduction of remaining Pd^*δ*+^ species [42, 43]. A clear difference between PdNRX and PdClRX catalysts is that the former has a negative peak at 326 K, which can be attributed to the decomposition of the *β*-PdH phase formed from metallic palladium during the reduction of Pd oxide species or during the catalyst preparation step at low temperatures [36]. Furthermore, for the PtClRX catalyst, two reduction peaks can be observed at approximately 479 and 555 K. According to other authors, both peaks could be associated with the reduction of Pt oxygenated species on the carbon surface [41, 44]. On the other hand, the RuClRX profile showed two peaks of hydrogen consumption during the TPR test: one at 450 K of high intensity and another one at 560 K of low area. Gómez-Sainero et al. [45] reported similar values for Ru catalysts supported on carbon, assigning both peaks to the reduction of ruthenium species according to the following mechanism: Ru^3+^ → Ru^2+^ → Ru°. Other authors [35] have assigned these signals to the reduction of ruthenium oxide and ruthenium chloride species.

Taking into account the XPS and TPR results (presented in Table 1 and Figure 3) it can be said that at the reduction temperature used during the preparation step, 673 K, the metal on each catalyst is totally reduced.

**Figure 4:**
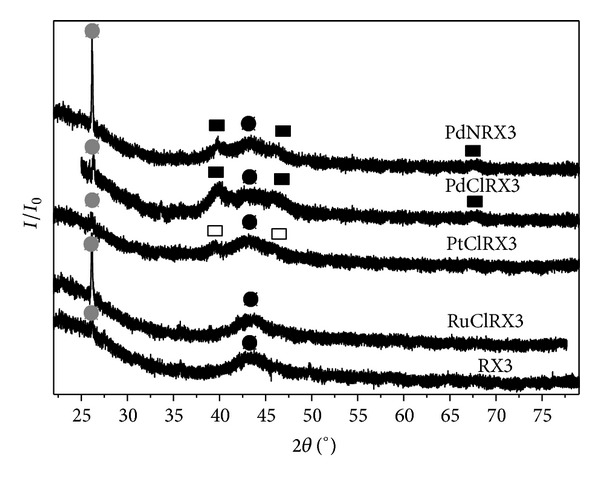
Diffractograms for PdClRX, PdNRX, PtClRX, and RuClRX catalysts and RX3 support (black square: Pd, white square: Pt, black circle: RX3, and grey circle: graphite).

The X-ray diffractograms of the PdClRX, PdNRX, PtClRX, and RuClRX samples are shown in Figure 4. The support diffractogram is also presented in Figure 4 in a comparative way, presenting two peaks at 2*θ* = 43.3° and 26.4°, being the former characteristic of the support while the last peak can be attributed to the presence of graphite crystalline structure [46, 47]. In Figure 4, the intensity of the graphite crystalline structure is bigger for PdNRX and RuClRX catalysts. Differences observed in the intensity of the graphite peak, in the PdClRX and PdNRX catalysts, are mainly due to the use of HNO_3_ during the preparation of PdNRX that produces not only the oxidation of carbon surface but also the formation of a crystalline carbon phase. This effect was also observed by other authors [31]. Both palladium catalysts showed three peaks at 2*θ* = 39.9°, 46.4°, and 68.4° that were assigned to Pd° [48]. Pinna et al. [35] reported similar results for Pd on carbon catalysts. Between both diffractograms there is a difference in the intensity of the peak at 2*θ* = 26.4°, which is assigned to the carbon in its crystalline structure of graphite. This peak is more intense for PdNRX, possibly because it comes from the reaction of HNO_3_ (it is present in the precursor solution) as an oxidant agent with the carbonaceous surface groups [49, 50].

In the diffractogram of the carbonaceous supported platinum catalyst (PtClRX), two peaks can be seen at 2*θ* = 39.9° and 46.2°, which are characteristic of the crystalline structures of Pt (111) and Pt (200), respectively [46]. For the Ru catalyst, no peaks are observed, possibly due to the proximity of a main peak characteristic of the crystalline phase of Ru (101) with the support peak at 2*θ* = 43.3°, as was also observed by other authors [33]. Anyhow, small particles of Ru on the catalyst as observed by TEM, well below the detection limit of this technique, are undetectable.

**Figure 5:**
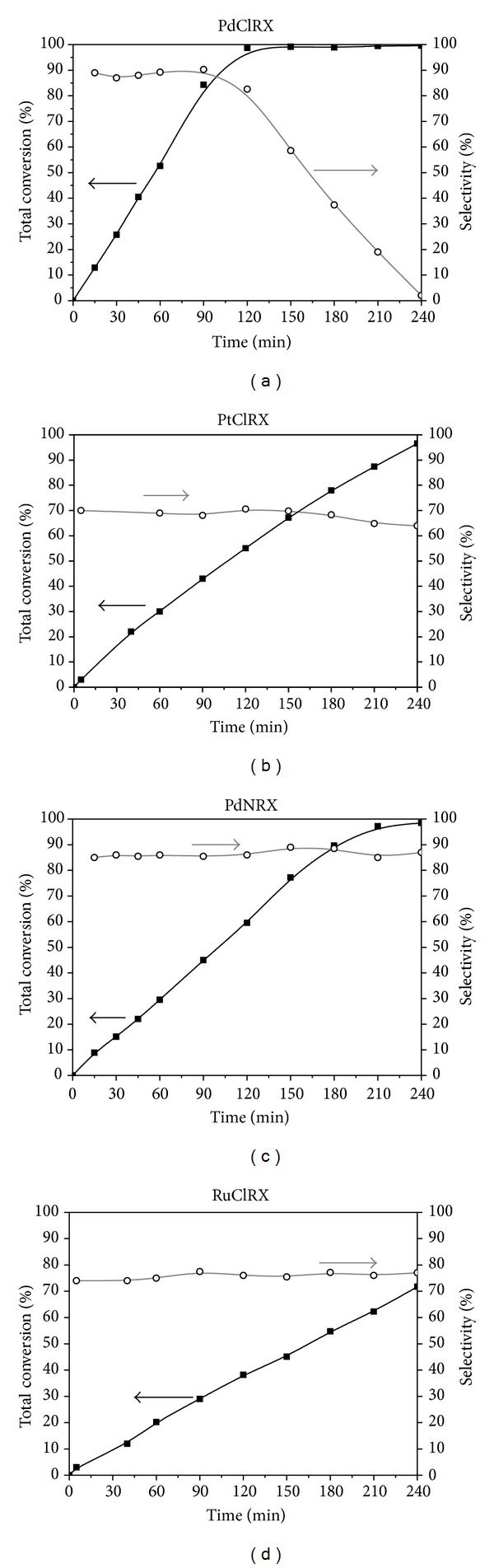
Total conversion and selectivity to 1-heptene as a function of time during 1-heptyne semihydrogenation using PdClRX, PdNRX, PtClRX, and RuClRX as catalysts. Temperature: 303 K; pressure: 150 kPa; catalyst mass: 0.75 g; 5% (v/v) 1-heptyne in toluene.

Total conversion and selectivity to 1-heptene as a function of time, obtained during the semihydrogenation of 1-heptyne, are presented in Figure 5. All catalysts are active in the hydrogenation of the carbon-carbon triple bond. The highest total conversion of the alkyne was obtained with the palladium catalyst prepared with the chloride precursor. The total conversion order of the reactant was the follows: PdClRX > PdNRX > PtClRX ≫ RuClRX. While there is 1-heptyne in the reacting media, both palladium catalysts have similar selectivity values, being the most selective catalysts under the studied reaction conditions. RuClRX and PtClRX were the least selective catalysts.

It is important to highlight the high hydrogenating capacity of Pd, with high selectivity values, compared to the other metals of group VIII, during the alkyne hydrogenation reactions. The high conversion is related to the percentage of d metal character. It can be rationalized that the hydrogen bond cleavage is more easily performed on the metal with the highest amount of electrons in the d orbital, as Pd, and more difficult on the metal with the smallest amount of electrons, like Ru [51]. Other authors have found similar results for acetylene hydrogenation [52–54] and for styrene selective hydrogenation [55]. However, it cannot be attributed only to electronic effects; it is possible that geometric factors play an important role since the activated carbon catalysts are rather complex from the point of view of porosity and surface chemistry.

The selectivity differences between the catalysts could be related mainly to the electron affinity of the metals (Pd < Ru < Pt) that favors the chemisorption of 1-heptyne thus promoting its total hydrogenation to n-heptane. This is associated with the differences in the adsorption-desorption equilibrium between the triple and double bond compounds and its competition for the active sites. The higher electron affinity produces the complete hydrogenation of the alkyne to the corresponding alkane, decreasing the selectivity of the catalytic system. These results are in total accordance with the fact that the alkene selectivity has a thermodynamic nature [56], directly related to the electronic affinity of the metals used. 

Our results thus suggest that the conversion and selectivity of palladium supported catalysts are a complex property of the whole catalyst and cannot be related to a single parameter. According to this, the slightly higher selectivity to 1-heptene found for PdClRX could be a consequence of a shape selectivity induced by the porous supports. This might be because the 1-heptene molecule has a planar end, unlike the more bulky end of fully saturated heptane. If the active Pd species are located in narrow pores, the formation of heptane could be hindered, thus increasing the selectivity to 1-heptene.

It is very important to know that the support has influence on the physicochemical properties and on the catalytic behavior of the metals [57]. The support specific properties, such as chemical composition, texture, and porous structure, can modify in different ways the morphology and/or distribution of metallic particles, the electronic structure of the superficial metal atoms, the adsorption-desorption equilibrium of the reactants, and so forth. Thus, if the support is modified during the preparation of the catalyst, it is possible to find different values of selectivity and total conversion. This is due to the complex properties of each catalyst, which are not related to a single parameter.

The change of precursor during the preparation of palladium catalysts does not alter significantly the selectivity to 1-heptene during the hydrogenation of 1-heptyne. Nevertheless, the highest total conversion is observed for PdClRX. The difference in activity between both palladium catalysts could be attributed to a better accessibility to the active sites in the chloride catalyst, due to the modification of the surface groups of the support as observed by the TPR technique.

## 4. Conclusions

Palladium, platinum, and ruthenium monometallic catalysts supported on an activated carbon were prepared. Chloride precursors of each metal and a palladium nitrate precursor were used to study the metal and precursor effects on activity and selectivity. Selective hydrogenation of 1-heptyne to 1-heptene was used as the test reaction.

TPR and XPS techniques suggest that the metals on the catalysts are totally reduced after the reduction treatment employed. The PdNRX TPR trace shows a notable modification of the support surface groups resulting from the use of HNO_3_ during the catalyst preparation step. 

During the partial hydrogenation of 1-heptyne to 1-heptene, palladium catalysts proved to be more active and selective than the platinum and ruthenium catalysts. The activity order found was as follows: PdClRX > PdNRX > PtClRX ≫ RuClRX. It can be rationalized that the hydrogen bond cleavage is more easily performed on the metal with the highest amount of electrons in the external d orbital (Pd) and more difficultly on the metal with the smallest number of d electrons (Ru). The different activities displayed by the catalysts could be partly attributed to a different electronic density in the external d orbital of the metal. Besides, the difference in activity between PdClRX and PdNRX catalysts could be assigned to a better accessibility of the alkyne to the active sites in the chlorinated catalyst. 

The selectivity order for the desired product (1-heptene) was as follows: PdClRX = PdNRX > RuClRX > PtClRX. These differences are attributed to thermodynamic factors of electron affinity differences between the metal active sites.

The surface groups of the activated carbon support are responsible for electronic and steric effects during the hydrogenation reaction.

## Figures and Tables

**Table 1:** Metal loadings, BET surface area, particle size (*d*), and XPS results.

Sample	M-loading (wt %)	*S*_BET_ (m^2^ g^−1^)	*d* (nm)	XPS BE (eV)	
RX3	—	1398	—	—	
PdClRX	1.62	1066	2.1	Pd 3d_5/2_	335.3
PdNRX	1.38	1063	3.3–5.3	Pd 3d_5/2_	334.9
PtClRX	1.71	1073	13.8	Pt 4f_7/2_	70.8
RuClRX	1.28	1158	0.80	Ru 3d_5/2_	280.0

M: metal Pd, Pt, or Ru.
